# Timing of Debridement in Low-Grade Open Forearm Fractures Does Not Affect Infection Risk: A Retrospective Study

**DOI:** 10.3390/jcm14092878

**Published:** 2025-04-22

**Authors:** Dani Rotman, Franck Atlan, Katherine Shehadeh, Itay Ashkenazi, Ron Gurel, Yishai Rosenblatt, Tamir Pritsch, Shai Factor

**Affiliations:** 1 Department of Orthopedic Surgery, Laniado Hospital, Adelson School of Medicine, Ariel University, Ariel 4070000, Israel; 2 Tel Aviv Medical Center, Department of Orthopedic Surgery, Faculty of Medicine, Tel Aviv University, Weizmann St 6, Tel Aviv-Yafo 6423906, Israel

**Keywords:** open fractures, Gustilo classification, forearm, infection rates, antibiotic therapy

## Abstract

**Background**: The timing of operative debridement for open upper extremity fractures has not been consistently shown to impact infection rates. Nevertheless, current treatment protocols continue to advocate for prompt surgical debridement in the operating room. We hypothesized that delaying the surgical treatment of low-grade open forearm fractures beyond 24 h from presentation does not increase the likelihood of infection. **Methods**: The medical charts of patients who presented to a level one trauma center with Gustilo type 1 or 2 open forearm fractures between 2017 and 2020 were retrospectively reviewed. Treatment protocols for these low-grade open fractures included prompt wound irrigation in the emergency department and intravenous antibiotic treatment for 72 h, without emphasizing the timing of surgical intervention. Outcome measures included time to surgery, infection rate, and union rate. **Results**: The mean ± standard deviation age of the 62-patient cohort was 57 ± 20 years, and 30 (48%) were males. There were 9 proximal third, 16 midshaft, and 37 distal third fractures, of which 41 involved both bones. Forty-eight fractures were classified as Gustilo type 1 and fourteen as Gustilo type 2. Surgery was performed at a median interval of 47 h following presentation, with 43 (69%) patients undergoing surgery later than 24 h following presentation. There was one case (1.6%) of infection and three cases (4.8%) of non-union. **Conclusions**: Subject to small numbers, our findings suggest that in patients without risk factors, surgical treatment for low-grade open forearm fractures can be safely deferred without an apparent increase in infection rates. Accordingly, treatment protocols for these fractures may prioritize prompt and adequate antibiotic administration over the urgency of surgical intervention.

## 1. Introduction

Open fractures account for approximately 6% of upper extremity fractures that require surgical intervention [1]. These fractures present an increased risk of infection compared to closed fractures, primarily due to the presence of associated soft tissue injuries and the potential for the contamination of deep structures. Despite these concerns, treatment guidelines for open fractures in the upper extremity remain largely extrapolated from the literature on lower extremity fractures, particularly open tibial fractures.

The unique anatomical and vascular characteristics of the upper limb, such as richer soft tissue coverage and a more robust blood supply, are believed to contribute to the relatively lower infection rates seen in open fractures of the upper extremity compared to the lower limb. Moreover, differences in biomechanical load-bearing and the wound environment further distinguish the clinical course and healing potential of these injuries [2].

Epidemiological studies suggest that upper extremity open fractures have lower infection rates than those of the lower extremities, with a large meta-analysis demonstrating a risk ratio of 0.51 (95% confidence interval 0.38–0.70) for developing a postoperative infection following upper extremity open fractures, relative to all open fractures [3]. Specifically, with regard to open fractures of the forearm, the limited available studies report a postoperative infection incidence ranging from 0% to 7% [1].

This variability in reported infection rates likely reflects heterogeneity in study populations, injury mechanisms, and institutional treatment protocols, underscoring the need for more granular, fracture-specific research on the upper extremity. This relatively low infection rate has influenced treatment paradigms, particularly for lower-grade open fractures. Several studies have shown that low-grade (Gustilo types 1 and 2) open forearm fractures can be successfully treated by prompt irrigation and debridement (I&D) coupled with open reduction and internal fixation (ORIF) during the same operation [4,5,6,7]. In these studies, patients presenting with open fractures of the radial and ulnar shafts or the distal radius underwent a single-stage surgical approach incorporating both I&D and fracture fixation, with outcomes comparable to those observed in patients with equivalent closed fractures. Furthermore, recent studies have demonstrated that the timing of the I&D of upper extremity fractures probably has only a small, if any, effect on the infection rate [1,8,9]. In the largest of these studies, 1298 cases of open upper extremity fractures were analyzed. In 79.7% of cases, surgery was performed on the day of hospital arrival, while the remaining cases experienced some degree of surgical delay. Notably, the infection rate did not differ significantly between the two cohorts (1.8% for early intervention vs. 1.1% for delayed surgery; *p* = 0.431) [1].

This finding challenges the longstanding surgical dictum of the “six-hour rule”, which historically advocated for emergent debridement to minimize infection risk. Although this concept remains embedded in many institutional guidelines, more recent evidence suggests that, particularly for low-grade injuries, modest delays in operative management do not compromise outcomes when appropriate antibiotic prophylaxis is administered early [9,10]. Despite this growing body of evidence, current clinical practice continues to favor early I&D in the operating room (OR), with or without ORIF, for all open fractures. Recommended treatments for open upper extremity fractures include the following: (1) primary irrigation and prompt administration of antibiotic therapy in the emergency department (ED); (2) continued antibiotic treatment for a duration of 24–72 h; and (3) urgent surgical intervention, generally within 6 to 24 h, for I&D and fracture fixation [11]. At our medical institution, the treatment protocol for low-grade open forearm fractures prioritizes immediate wound irrigation in the ED, combined with intravenous antibiotic administration for 72 h. Whenever feasible, surgical intervention—including I&D and definitive fracture fixation via ORIF—is expedited. However, in certain cases, surgical treatment is postponed due to factors such as OR scheduling constraints or the need for medical optimization of the patient’s condition.

Given the logistical and clinical factors that may delay surgical management, clarifying whether such postponements meaningfully impact infection outcomes is of considerable practical relevance. Despite the abundance of literature on open fractures in general, there remains a notable lack of focused data addressing the timing of debridement specifically for low-grade open forearm fractures, leaving clinicians with limited evidence to guide decision-making in this common scenario. Based on these considerations, we hypothesize that delayed surgical treatment (beyond 24 h from initial presentation) for low-grade open forearm fractures does not result in an increased risk of infection.

## 2. Materials and Methods

This study was approved by our institutional review board. We retrospectively reviewed the medical charts of all patients over 18 years of age who presented with a low-grade (Gustilo and Anderson type 1 or 2) open forearm fracture to the ED of a single tertiary trauma center between January 2017 and March 2021. Excluded were patients who received initial treatment at another medical facility, elected to continue their treatment at another medical facility, or whose follow-up was less than six months In addition, patients with diabetes mellitus, immunosuppressive conditions, chronic alcohol use, an active smoking status, or other significant comorbidities that could influence the infection risk or the timing of surgical management were excluded from the study.

### 2.1. Treatment Protocol

Our treatment protocol for open upper extremity fractures includes the following steps:

(1) Upon presentation to the ED, patients receive prompt intravenous antibiotic treatment, including first-generation cephalosporin (2 g cefazolin) in all cases, with added aminoglycoside (240 mg gentamycin) for Gustilo type 2 or 3 fractures. Patients allergic to cephalosporins receive 900 mg of clindamycin instead. The tetanus toxoid status of all patients is checked, and a tetanus toxoid shot is administered when indicated;
(2) Following initial clinical assessment and the performance of radiographs, the wound is irrigated in the ED, and the fracture is temporarily fixated using a plaster splint;
(3) Patients with Gustilo type 3 open fractures are taken directly from the ED to the OR for a formal I&D and initial fracture fixation, usually by external fixation;
(4) All patients are hospitalized and receive intravenous antibiotic treatment for 72 h, regardless of the time to surgery: first-generation cephalosporin (1 g cefazolin three times per day) in all cases, with added aminoglycoside (240 mg gentamycin once daily) in Gustilo type 2 or 3 fractures. Patients allergic to cephalosporins receive 600 mg of clindamycin three times per day instead of cefazolin. Patients who refuse hospitalization are discharged with the recommended 72 h of oral antibiotic treatment, consisting of 1 g cephalexin three times per day;
(5) Patients with Gustilo type 1 or 2 open fractures are taken to the OR at the next available session for I&D and definitive fracture treatment by ORIF with a locking plate at the same session. In cases where surgical treatment is delayed for more than 72 h, patients are discharged from the hospital and return to undergo surgical treatment as outpatients;
(6) If surgery is performed after the patients have completed their course of antibiotic treatment, no further antibiotic treatment is routinely administered following surgery, unless the surgeon decides otherwise based on intraoperative findings (e.g., poor soft tissue quality);
(7) Follow-up visits are scheduled at two weeks, six weeks, three months, six months, and one year, and all visits include updated radiographs.

### 2.2. Data Collection

The medical charts of all study participants were reviewed, and their demographic data were recorded. An attempt was made to contact patients who discontinued their follow-up by telephone to inquire about any symptoms suggestive of fracture non-union or infection, including pain, redness, swelling, and drainage. Fracture patterns were retrospectively classified according to the AO/OTA classification [12] by one of the senior authors. The Gustilo and Anderson open fracture classification was determined by the treating physician in the ED [13]. Outcome measures included time to surgery, infection rate, and union rate, as evidenced both clinically and radiographically. Postoperative infection was defined as the presence of clinical signs including erythema, warmth, swelling, local tenderness, wound drainage (purulent or persistent serous), or wound dehiscence, with or without accompanying systemic signs such as fever. Diagnosis was supported by the treating surgeon’s clinical judgment and, when available, corroborated by elevated inflammatory markers (e.g., C-reactive protein, leukocytosis) and/or positive wound or blood cultures.

Non-union was defined as the failure of radiographic healing by 6 months postoperatively, as evidenced by persistent fracture lines on plain radiographs in at least three out of four cortices in anteroposterior and lateral views, in conjunction with clinical symptoms such as pain at the fracture site or the absence of functional recovery. The final diagnosis was determined by the attending orthopedic surgeon based on both clinical and radiographic criteria.

### 2.3. Statistical Analysis

Continuous variables are presented as mean (±standard deviation [SD]) if normally distributed or median (interquartile range [IQR]) if not. Due to the low infection rate recorded among the study participants, no regression analysis was performed to examine the effect of surgical timing on the occurrence of fracture-associated infection.

## 3. Results

**Figure 1:**
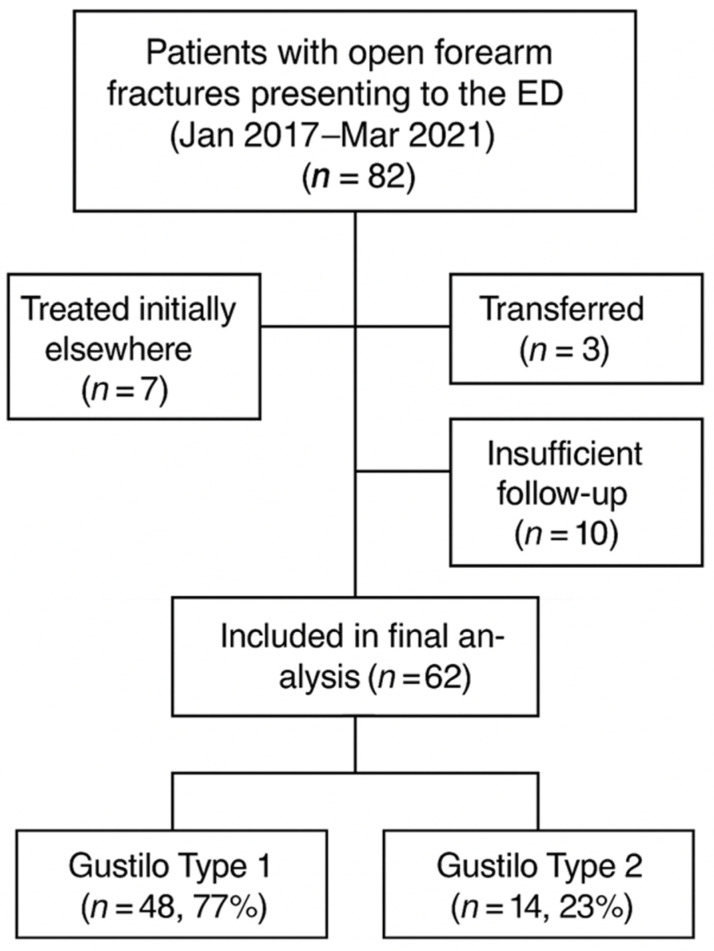
Study inclusion and classification of patients by Gustilo type.

Eighty-two patients with low-grade open forearm fractures presented to the ED during the study period. Twenty of them were excluded (7 had been initially treated at another hospital, 3 transferred to another institution for further treatment, and 10 lacked a sufficient follow-up), leaving 62 cases (76%) in our study group (Table 1, Figure 1).

The cohort’s mean age was 57 ± 20 years, and 30 (48%) were males. There were 9 proximal third forearm fractures (3 involving both bones and 6 involving the olecranon); 16 midshaft forearm fractures (14 involving both bones and 2 involving the ulna), of which 3 were periprosthetic fractures adjacent to a previously placed plate; and 37 distal third fractures (24 involving both bones and 13 involving the distal radius). The fracture pattern according to the AO/OTA classification is detailed in Table 2. Forty-eight cases were classified as Gustilo type 1, and fourteen as Gustilo type 2. The mean follow-up was 24 ± 14 months (range 7–59 months). Surgical treatment was provided at a median time of 47 h following presentation to the ED (IQR 22 h to 4 days, range 1 h to 11 days). Fifteen patients (24%) were operated on within 24 h, and 43 patients (69%) later than 24 h. Four patients (6%) refused surgical treatment and were treated conservatively by IV antibiotics and cast immobilization, all with a Gustilo type 1 open fracture. Six patients (10%) refused hospitalization for IV antibiotics following the initial presentation and were discharged from the ED with oral antibiotics, all with a Gustilo type 1 open fracture.

There was one (1.6%) case of a deep wound infection in a patient with a Gustilo type 2 open fracture. The infection had already been present during the surgery, which was performed 53 h following presentation. There were three (4.8%) cases of non-union following ORIF, one of them the infected case. Two patients underwent revision ORIF surgery with bone grafting at 3 and 8 months following the original ORIF, with complete union at final follow-up. The third case is scheduled to undergo revision of the ORIF in the near future.

## 4. Discussion

This study suggests that in Gustilo type 1 and 2 open forearm fractures, a treatment protocol involving prompt irrigation and intravenous antibiotics in the emergency department, followed by delayed but definitive surgical fixation, is associated with low infection (1.6%) and non-union (4.8%) rates. These outcomes are comparable to those reported in large series of surgically treated closed forearm fractures [14,15]. For example, Vasara et al. found a 2% deep infection rate and 5% non-union rate in a cohort of 470 such cases [16].

These results hold clinical significance, as they suggest that, unlike open fractures of the lower extremity, low-grade open fractures of the forearm do not necessitate urgent surgical intervention in the OR [17]. Indeed, a substantial proportion (76%) of our study patients underwent surgery beyond 24 h following the initial presentation, and 50% were after 47 h, with no corresponding increase in infection incidence. Based on these data, we propose that current treatment guidelines for low-grade open forearm fractures should be re-evaluated to emphasize the importance of timely and adequate antibiotic administration rather than rigid adherence to emergent surgical debridement.

Deferring surgical intervention necessitates a reliable and thorough initial evaluation in the ED. Current consensus guidelines advocate for urgent operative debridement of all open fractures, with intraoperative findings serving as the definitive basis for Gustilo and Anderson classification [13]. However, our findings raise questions regarding the necessity of this approach. Notably, the only patient in our cohort who developed an infection was subsequently found to have periosteal stripping of the ulna and extensive soft-tissue contamination characteristics consistent with a Gustilo type III open fracture. In this instance, we relied on the original ED-based classification, as this represents the primary information available to the senior on-call orthopedic surgeon when determining surgical urgency. Misclassification remains an inherent limitation of ED-based assessments, particularly for “borderline” Gustilo type II fractures. To mitigate this issue, our institution has implemented a standardized protocol incorporating wound photography at initial assessment, providing the on-call senior surgeon with additional information to aid in surgical decision-making.

The existing literature assessing the timing of surgical intervention for open forearm fractures is heterogeneous, with many studies defining “delayed” surgery as exceeding 6 or 8 h [18]. Consequently, drawing definitive conclusions regarding longer surgical delays remains challenging (Table 3). That said, several studies have reported consistently low infection rates for low-grade open forearm fractures, irrespective of the time to surgery [19]. Zumsteg et al. retrospectively analyzed 200 cases of open forearm fractures, 89 of which were classified as low-grade (Gustilo type I or II) [19]. Notably, none of the 41 Gustilo type I cases and only 2 of the 48 Gustilo type II cases (4%) developed infections, despite a mean surgical delay of 16 h. Their findings did not demonstrate an association between surgical delays exceeding 6 h and increased risks of infection or non-union.

Conversely, Rust et al. reported a single-center case series of ORIF for both-bone forearm fractures. Unlike previous studies, their findings suggested that both the presence of an open fracture (compared with closed fractures) and surgical delays exceeding 48 h (compared with earlier intervention) were associated with an increased risk of complications. These findings highlight the ongoing debate regarding the optimal surgical timing for open forearm fractures [20].

The literature about open distal radius fractures is more robust and supports our findings: Kurylo et al. reported 32 cases of open distal radius fractures, of which 29 were low-grade (Gustilo type 1 and 2). None of the cases in their series developed an infection, leading the authors to conclude that neither the time to surgical debridement nor the method of initial fixation significantly influenced infection risk [21]. Similarly, Morrison et al. conducted a retrospective review of 120 patients treated for open distal radius fractures. Among these, 24 patients (20%) underwent surgery more than 24 h post-presentation, with outcomes that were not significantly different from those who received an earlier intervention [22].

Colello et al. investigated 62 patients who underwent surgical treatment for Gustilo type I open distal radius fractures. Among them, 38 underwent surgery within 24 h, while 24 had a delayed surgery (mean delay: 72 h). Their cohort exhibited only a single infection case, which occurred in the early surgery group. Based on these results, the authors concluded that Gustilo type I open distal radius fractures could be safely treated beyond 24 h post-injury without an elevated risk of infection [23].

Recently, Nemirov et al. published the largest series to date on open distal radius fractures, including 230 cases. Their cohort comprised 40% Gustilo type I, 40% type II, and 20% type III fractures. The authors found no significant difference in outcomes between patients treated within 24 h and those treated later, with a deep infection rate of 6.5% and no reported cases of radiographic non-union [24].

Although we suspect that delaying surgical treatment for low-grade upper extremity fractures is common practice, we did not identify published data to substantiate this assumption. In a large-scale retrospective study, Ryan et al. [1] examined 22,578 upper extremity fractures, 1298 (5.7%) of which were open fractures. In their study, the majority (79.7%) of patients with open fractures underwent early surgical intervention; however, infection rates did not differ between those receiving immediate versus delayed surgery. The authors hypothesized that fractures successfully managed with delayed surgery were likely lower-grade injuries, which inherently carry a lower risk of infection.

Although midshaft forearm fractures carry a theoretical risk of developing acute compartment syndrome (ACS), no cases of ACS were observed in our cohort. At our institution, patients are monitored closely during their stay in the emergency department and inpatient unit by both experienced nursing staff and the orthopedic team, with multiple evaluations per day. There is a high level of awareness regarding the clinical signs of evolving ACS, particularly disproportionate pain, which is typically the earliest and most sensitive indicator.

A recent systematic review by AlHussain et al. [25] emphasized that fractures, especially both-bone fractures of the radius and ulna, are among the most significant risk factors for the development of ACS. The review further highlighted that while open fractures may intuitively seem to reduce compartmental pressure, they nonetheless remain a potent cause of ACS. Still, the incidence remains low, particularly when patients are monitored appropriately and managed in a timely manner. Our experience supports these findings, suggesting that with adequate clinical surveillance, surgical delays in the treatment of selected low-grade open midshaft forearm fractures can be justified without increasing the risk of compartment syndrome.

This study has several limitations. The most significant is the small sample size, which limits its statistical power and the ability to detect meaningful differences in infection risk between groups. While subgroup analyses were performed, the limited number of events, particularly in subgroups such as Gustilo type 2 fractures and infection cases, restricts the ability to detect smaller, yet clinically relevant, differences. As a result, this study is underpowered to identify anything but very large differences in infection risk. Additionally, the study cohort consisted primarily of relatively healthy individuals, with exclusion criteria that eliminated patients with comorbidities known to influence infection risk, such as diabetes, smoking, and immunosuppression. Although this exclusion helped ensure a more homogenous study population, it also means that the findings may not be generalizable to patients with higher-risk comorbidities, who may experience different outcomes. Finally, the retrospective design of the study introduces potential biases, and the absence of a direct comparative analysis between urgent and delayed surgical intervention further limits the ability to draw firm conclusions regarding the optimal timing of surgery. Another important limitation is the absence of a dedicated analysis evaluating the impact of antibiotic timing on infection outcomes. While all patients received intravenous antibiotics promptly upon arrival to the emergency department, according to a standardized institutional protocol, the exact timing from injury or presentation to antibiotic administration was not independently analyzed. Therefore, while the consistency in initial management strengthens the internal validity of our findings, we cannot definitively isolate the effect of antibiotic timing from other factors influencing infection risk. Given these limitations, caution should be taken when interpreting the results, and further prospective studies with larger sample sizes are necessary to validate these findings in a broader patient population.

Despite these limitations, this study has notable strengths. The investigation was focused on a well-defined patient population, low-grade open forearm fractures, allowing for a more controlled analysis. Furthermore, the consistency in initial treatment protocols in the ED enhances the reliability of our findings.

**Table 3:** Recent research on open forearm fractures and treatment approaches.

	Year	Sample Size	Study Design	Key Findings
Rust et al. [20]	2024	99 patients	Retrospective study assessing timing of surgery on union rates	Surgery delayed >48 h associated with increased delayed union (59% vs. 25%, *p* = 0.03); no significant difference in complications
Luo et al. [26]	2023	20 patients	Retrospective study on Gustilo IIIB fractures with free flap coverage	Limb salvage successful in all cases; 15% superficial infection rate; no deep infections or osteomyelitis
Shu et al. [27]	2023	65 patients	Retrospective analysis; delayed wound closure more common in high-energy and open fractures	20% underwent delayed wound closure; male gender was independently associated with increased odds (OR 9.9, *p* = 0.04)
Harper et el. [7]	2020	90 patients	Retrospective review focusing on open distal radius fractures	Immediate ORIF resulted in satisfactory outcomes; 37% complication rate observed
Ahmad et al. [28]	2020	29 patients (12 open)	Prospective cohort comparing outcomes in open vs. closed diaphyseal fractures	At 6 weeks, better ROM in closed fractures (83% vs. 27%, *p* = 0.01); no significant difference beyond 3 months

## 5. Conclusions

Low-grade open forearm fractures (Gustilo type I or II) may be managed effectively with early intravenous antibiotics and thorough wound irrigation in the emergency department, followed by a delayed, planned surgical intervention. In this retrospective cohort, delayed surgery beyond 24 h was not associated with a higher rate of infection or non-union. These findings support a treatment approach that prioritizes timely antibiotic administration and appropriate initial wound care, while allowing flexibility in surgical scheduling. This strategy may help optimize resource allocation without compromising patient outcomes.

## Figures and Tables

**Table 1:** Patients’ demographics.

**Total patients screened**	82
**Excluded patients**	20 (7 treated elsewhere, 3 transferred, 10 lacked follow-up)
**Study cohort**	62 (76% of total)
**Mean age, years,** ±**SD**	57 ± 20
**Gender (%)**	
males	30 (48%)
females	32 (52%)
**Fracture location (%)**	
- Proximal third	9 (15%)
- Midshaft	16 (26%)
- Distal third	37 (60%)
**Fracture type, Gustilo (%)**	
1	48 (77%)
2	14 (23%)
**Follow-up, months** ± **SD**	24 ± 14 (range 7–59)
**Median time to surgery**	47 h (range 1 h–11 days)
**Surgery timing (%)**	
within 24 h	15 (24%)
>24 h	43 (69%)
**Conservative management (%)**	4 (6%)
**Oral antibiotics only (%)**	6 (10%)
**Deep wound infection (%)**	1 (2%)
**Non-union cases (%)**	3 (5%)

SD; Standard deviation.

**Table 2:** Fracture morphology distribution according to AO\OTA classification.

Location	Proximal		Midshaft		Distal	
	Both	Ulna Only	Both	Ulna Only	Both	Radius Only
A		2	8 *	2 *	13	5
B	2	2	5		1	
C	1	2	1		10	8

* Periprosthetic fractures refer to new traumatic fractures occurring adjacent to pre-existing forearm hardware from previous fracture fixation.

## Data Availability

The data presented in this study are available on request from the corresponding author due to ethical restrictions.
